# Behavioral Responses of *Chrysoperla defreitasi* (Neuroptera: Chrysopidae) and *Myzus persicae* (Hemiptera: Aphididae) to Volatile Compounds from Wild and Domesticated *Ugni molinae*

**DOI:** 10.3390/insects16060594

**Published:** 2025-06-05

**Authors:** Manuel Chacón-Fuentes, Leonardo Bardehle, César Burgos-Díaz, Marcelo Lizama, Daniel Martínez-Cisterna, Mauricio Opazo-Navarrete, Cristina Bravo-Reyes, Andrés Quiroz

**Affiliations:** 1 Agriaquaculture Nutritional Genomic Center, CGNA, Temuco 4780000, Chile; cesar.burgos@cgna.cl (C.B.-D.); mauricio.opazo@cgna.cl (M.O.-N.); 2 Laboratorio de Química Ecológica, Departamento de Ciencias Químicas y Recursos Naturales, Universidad de La Frontera, Av. Francisco Salazar 01145, Casilla 54-D, Temuco 4811230, Chile; leonardo.bardehle@ufrontera.cl; 3 Centro de Investigación Biotecnológica Aplicada al Medio Ambiente (CIBAMA), Universidad de La Frontera, Av. Francisco Salazar 01145, Casilla 54-D, Temuco 4811230, Chile; 4 Departamento de Producción Agropecuaria, Facultad de Ciencias Agropecuarias y Medioambiente, Universidad de La Frontera, Av. Francisco Salazar 01145, Casilla 54-D, Temuco 4811230, Chile; 5 Programa de Doctorado en Ciencias Agroalimentarias y Medioambiente, Facultad de Ciencias Agropecuarias y Medioambiente, Universidad de La Frontera, Temuco 4811230, Chile; marcelolizamavera@gmail.com; 6 Programa de Doctorado en Ciencias de Recursos Naturales, Facultad de Ingeniería y Ciencias, Universidad de La Frontera, Temuco 4811230, Chile; d.martinez11@ufromail.cl; 7 FoodTech SpA, Temuco 4780000, Chile; contacto@foodandtech.cl

**Keywords:** insect–plant interactions, lacewing, aphid, VOCs, plant domestication, chemical ecology

## Abstract

Domestication significantly altered the phenotypic and chemical traits of murtilla, notably reducing the emission of volatile compounds essential for plant–insect interactions. This reduction may affect the plant’s natural defense mechanisms, influencing its interactions with herbivores and predators. Therefore, this study tests whether domestication reduces volatile emissions in murtilla, increasing aphid preference and decreasing lacewing attraction. We selected wild ancestors (19-1, 22-1, and 23-2) from a longterm *Ugni molinae* germplasm bank. Crosses between these wild ancestors generated four first-generation domesticated ecotypes, 10-1, 16-16, 17-4, and 66-2, used in this study. These first-generation domesticated ecotypes were six years old at the time of the study and were used for comparisons in volatile profile and insect interaction analyses. The olfactometric preference index (OPI) for lacewing larvae and aphids revealed that wild ancestors attracted more predators than domesticated plants. For example, Ecotype 19-1 had an OPI of 1.64 for larvae and 1.49 for aphids, while Ecotype 10-1 showed lower attraction (OPI of 1.01 for larvae and 1.00 for aphids). Gas chromatography analysis identified differences in volatile organic compounds, with wild ancestor ecotypes emitting higher levels of compounds such as 2-hexanone, 1,8-cineole, and α-caryophyllene. Principal component analysis and hierarchical clustering confirmed these chemical distinctions. In olfactometer assays, lacewing larvae preferred α-caryophyllene and 2,4-dimethyl acetophenone, while aphids favored 2-hexanone and 3-hexanol. In Y-tube assays, lacewing adults showed strong attraction to α-pinene and 2,4-dimethyl acetophenone, with preferences increasing with concentration. These results indicate that domestication altered the volatile murtilla profile, reducing its attractiveness to natural predators while increasing its susceptibility to herbivores, supporting the plant domestication defense theory.

## Simple Summary

Many crops changed through domestication to improve traits such as yield and fruit quality, but this process often weakens their natural defenses against pests. Murtilla, a berry native to Chile, has undergone similar changes, producing fewer natural chemicals that help protect it from insects. Wild murtilla plants release more of these compounds, which attract helpful predators, such as lacewing larvae, and deter harmful pests, such as aphids. In contrast, domesticated murtilla emits fewer defensive chemicals, making it more vulnerable to aphid infestations. In this study, we compared the chemical profiles of wild and domesticated plants and tested how insects responded to them. We found that wild plants emitted higher amounts of key protective compounds, while domesticated plants produced less. Experiments showed that lacewing larvae were more attracted to certain chemicals found in wild plants, while aphids preferred those present in domesticated ones. These findings suggest that domestication unintentionally made murtilla more susceptible to pests by weakening its natural defenses. Understanding these changes can help improve future agricultural practices, potentially leading to breeding strategies that balance fruit quality with natural resistance to pests, reducing the need for chemical pesticides.

## 1. Introduction

Plant domestication has been a pivotal process in the evolution of agriculture, transforming wild species into crops with traits better suited to human needs [1,2]. While this process resulted in higher yields, improved taste, and larger fruit size, it also caused a reduction in natural defenses against herbivores and pathogens [3,4]. This phenomenon, known as the domestication defense hypothesis, suggests that cultivated plants invest less in chemical defenses, making them more vulnerable to pests [5,6]. Understanding how domestication influences plant–insect interactions is crucial for developing sustainable agricultural practices that reduce reliance on chemical pesticides. Plants primarily defend themselves by producing volatile organic compounds (VOCs), which play key roles in plant–insect communication [7]. These compounds attract natural predators of herbivores or directly repel pests. Numerous studies have shown that wild plants produce higher levels of VOCs compared to their domesticated counterparts [5,6]. For example, wild tomato species emit more terpenes, which repel herbivorous insects, than cultivated varieties [8]. Similarly, wild cotton plants produce higher levels of toxic secondary metabolites that protect them from pests [9,10]. These findings highlight the ecological importance of VOCs in maintaining plant health and resilience. Consequently, domestication caused a reduction in the production of these defensive compounds, affecting the plants’ ability to deter herbivores and attract natural enemies through indirect defense mechanisms [11,12]. An interesting example of this phenomenon is murtilla (*Ugni molinae*), a native berry from Chile, which has become the subject of commercial cultivation efforts. Traditionally harvested from wild populations, murtilla has recently seen increased breeding for higher productivity. While much focus has been placed on enhancing its yield, limited information is available on how domestication affects its defensive chemicals and insect interactions [13,14,15]. Murtilla produces a range of VOCs, which play vital roles in mediating interactions with beneficial and harmful insects [16,17,18,19]. These compounds help the plant defend itself by either attracting natural predators of herbivores or repelling pests directly. However, it is suspected that domestication may reduce murtilla’s ability to produce these defensive VOCs, potentially making it more susceptible to pest infestations. Over the last 25 years, breeding programs primarily focused on enhancing productive traits, such as fruit yield and diameter, with secondary attention to antioxidant activity [20,21]. While these efforts improved yield and other desirable features, they also compromised the plant’s natural chemical defenses. For example, domestication has been shown to reduce the emission of key compounds such as 2-hexanone, 1,8-cineole, and α-caryophyllene, which are essential for defense responses and insect interactions. Chacón-Fuentes et al. [16] reported that these compounds decreased from detectable levels in wild plants (0.40%, 0.39%, and 0.57%, respectively) to undetectable levels in domesticated plants. This reduction in volatile emissions may influence herbivore preference, as demonstrated by aphids such as *Myzus persicae* (Hemiptera: Aphididae), which are less deterred by the reduced emissions in domesticated plants, making them more susceptible to infestations [22]. In addition, this decline in chemical defenses affects natural predators, influencing their ability to detect and respond to changes in the volatile profiles of the plant. For instance, lacewing larvae (*Chrysoperla defreitasi*, Neuroptera: Chrysopidae), which rely on VOCs to locate their prey, showed less attraction to domesticated plants compared to wild ones [23]. One major concern in crop domestication is the loss of natural resistance to herbivores. Often, cultivated plants attract more pests due to reduced production of defensive chemicals. For example, studies on domesticated maize have shown that it emits lower levels of certain VOCs that attract parasitoids of pest insects, making it more vulnerable to infestations [24]. Similarly, domesticated apple varieties produce fewer defensive compounds than their wild relatives, leading to increased herbivore damage [25]. If similar patterns occur in murtilla, domesticated ecotypes may face higher aphid infestations due to reduced volatile emissions, while wild plants may maintain stronger defenses. Natural predators, such as lacewings, play a key role in controlling pest populations [26,27]. Thus, if domesticated murtilla plants emit lower concentrations of these compounds, lacewing attraction may decrease, potentially leading to higher aphid populations. In contrast, wild murtilla plants may continue to emit higher levels of predator-attracting volatiles, providing better protection against herbivores.

*Ugni molinae* is an endemic Chilean shrub with increasing agronomic and commercial relevance. Despite its potential, the effects of domestication on its chemical defenses, particularly volatile organic compounds, remain largely unexplored. VOCs play a key role in mediating plant–insect interactions, including herbivore deterrence and predator attraction. While changes in VOC emissions due to domestication have been reported in crops such as tomato and cotton, there is a notable gap in understanding how such changes occur in *U. molinae*. This study addresses this gap by evaluating VOC emission differences between wild and domesticated murtilla and assessing their behavioral effects on aphids and a natural predator. Our findings may contribute to developing more sustainable pest management strategies in murtilla cultivation [28,29,30,31,32,33,34,35].

This study investigates how domestication affects VOC profiles in *U. molinae* and how these changes influence insect behavior. We hypothesize that domesticated ecotypes emit lower levels of defensive VOCs, increasing aphid preference (*M. persicae*) and reducing the attraction of natural predators such as *C. defreitasi*. This could disrupt ecological interactions and reduce biological control efficiency [36,37,38]. To test this, we analyzed VOC emissions from wild ancestors and their selected F1 progeny, previously underexplored compared to studies focusing on clonal propagation, and assessed insect responses. Our findings provide novel insights into how domestication alters plant–insect interactions in this endemic Chilean crop.

## 2. Materials and Methods

### 2.1. Plant Material and Insects

The selection of ecotypes for this study was based on their agronomic potential and biochemical properties. Wild ancestor ecotypes (19-1, 22-1, and 23-2) were chosen from a long-term germplasm bank established 25 years ago in Carahue, La Araucanía region, Chile. These ecotypes were selected due to their high fruit yield, larger fruit size, and elevated antioxidant content [5,15,39]. To evaluate the early stages of domestication, controlled crosses were performed between the selected wild ancestor ecotypes, generating first-generation (selected F1 progeny) hybrids corresponding to four domesticated ecotypes (10-1, 16-16, 17-4, and 66-2). These selected F1 progeny were designated as “domesticated” based on their status as the initial step in a selective breeding process aimed to enhance desirable traits. Importantly, all plants used in this study, including wild ancestor ecotypes and selected F1 progeny, were maintained under the same environmental and soil conditions in a common garden to ensure that differences in volatile profiles were attributable to genetic factors and not environmental variation. These selected F1 progeny were approximately six years old at the time of the study and reached full production under cultivation. For the experiments, samples of these plants were collected and transported to the Laboratory of Chemical Ecology at Universidad de La Frontera, where all subsequent analyses were conducted. This selection process allowed for a direct comparison between wild ancestor and domesticated ecotypes to assess how early selection influences plant volatile profiles and insect interactions (Table 1). For clarity, each ecotype was classified as either wild or domesticated. Throughout the text, they are referred to as Wild Ecotypes 1–3 (W1–W3) and Domesticated Ecotypes 1–4 (D1–D4). The correspondence between these labels and the original identifiers is provided in Table 1. All ecotypes were cultivated under uniform conditions in a common garden during the 2022–2023 season, with full sun exposure, sandy loam soil, drip irrigation every 48 h, and preventive pest management using biological inputs only (e.g., *Beauveria bassiana*). These standardized conditions minimized environmental variation, allowing us to attribute differences in volatile emissions mainly to genetic and domestication effects.

Lacewing larvae were purchased from Biobichos Ltda. (Chillán, Chile), a commercial insectary that maintained this species in continuous culture since 2011. The colony is reared under controlled conditions (25 ± 2 °C, 70–80% relative humidity, and a 16:8 h light:dark photoperiod), and wild individuals are periodically introduced to maintain genetic diversity and ecological performance. The insects arrived in dispensing boxes containing a rice husk substrate where the lacewing eggs were collected. After hatching, the larvae were reared in an acrylic chamber (30 × 30 × 30 cm). Lacewing larvae were fed *Sitotroga cerealella* (Lepidoptera: Gelechiidae) eggs and maintained under laboratory conditions (25 °C, 80% relative humidity, 16:8 h light:dark photoperiod) for acclimation. After three days of emergence, the larvae were individually separated into plastic pots (56 × 30 mm), where they remained until their use in the four-arm olfactometric assays.

Lacewing larvae not used in the assays were allowed to feed and pupate until they reached the adult stage. Adults were used immediately after emergence in Y-tube olfactometric assays. Aphids (*M. persicae*) were collected from rose plants in Temuco, Chile, and subsequently maintained on healthy rose plants (*Rosa* spp.) under controlled conditions (22 ± 1 °C, 60–70% relative humidity, and a 16:8 h light:dark photoperiod). Prior to the olfactometric assays, aphids were food-deprived for 24 h [16].

### 2.2. Volatile Collection System

Volatile compounds from whole plants were collected over a 24 h period (1 L/min) following the methodology reported by Chacón-Fuentes et al. [16]. Each plant was enclosed in a 900 mL glass chamber (8 cm in diameter and 30 cm in height). Volatiles were trapped using 200 mg Porapak-Q columns (80–100 mesh, Waters Associates, Milford, MA, USA), which were pre-cleaned with diethyl ether (GC grade, Merck, Darmstadt, Germany) and conditioned at 150 °C for two hours in a nitrogen stream (70 mL/min). The Porapak-Q was placed inside 10 cm-long and 1 cm-wide collection columns, with two columns per plant. The entrainment was performed using a positive/negative pressure air system as described by [5]. After collection, the volatile compounds were eluted with 1 mL of hexane (GC-MS grade, Optima Scientific, Darmstadt, Germany) and concentrated to 100 µL using a nitrogen flow. Subsequently, each obtained extract was resuspended in 1 mL of hexane to be used in the bioassays (see below). The plant material collected included leaves, stems, and branches (no plants were in bloom or fruiting), ensuring that a comprehensive sample of the plant’s volatile profile was captured. Volatile compound collection was performed using nine different plants per ecotype (n = 9), with each replicate collected from a different individual plant to ensure independent replication.

### 2.3. Gas Chromatography Coupled to Mass Spectrometry Analysis

The volatile compounds were analyzed using gas chromatography coupled with mass spectrometry (GC-MS) (Focus DSQ, Thermo Electron Corporation, Waltham, MA, USA). Data were acquired and processed using Xcalibur software, version 2.2 (Thermo Electron Corporation, Waltham, MA, USA). Separation was performed on a BP-1 capillary column (30 m × 0.22 mm × 0.25 µm) with helium as the carrier gas (1.0 mL/min). The initial temperature was set at 40 °C for 2 min, then increased by 5 °C/min until reaching 250 °C. The injector and interface temperatures were maintained at 250 °C, with the detector at 200 °C. Electron impact ionization energy was set at 70 eV, and the mass spectra were acquired over a 30–350 *m*/*z* mass range. Just 1 µL aliquot of volatiles was injected into the GC-MS for analysis. Retention time peaks were matched to the NIST mass spectrum library (Version 2.0) to verify compound identities. In addition, the compounds captured through the Porapaq Q, which were detected, were confirmed and quantified by comparison with their respective commercial standard (Merck, Darmstadt, Germany). Finally, to adjust VOC values, branch standardization was performed, incorporating corrections based on leaf surface area and stem diameter for each evaluated plant. Leaf area measurements were obtained by scanning all the leaves of each branch and analyzing them with ImageJ 1.42 J software (U.S. National Institutes of Health, Bethesda, MD, USA). The stem surface area was estimated using the geometric formula for a cylinder. This methodology allowed for a more accurate determination of the total surface area contributing to VOC emissions in each plant [5].

### 2.4. 4-Arm Olfactometric Assays

Olfactometric tests were performed using a four-arm Pettersson olfactometer (105 × 110 mm) to evaluate the behavioral response of *C. defreitasi* larvae and *M. persicae* aphids over a 30 min period [5]. This olfactometer was chosen for these insects and developmental stages due to the size of both the insects and the olfactometers, which are appropriately calibrated to ensure compatibility for the study, allowing for precise and efficient analysis without complications. Briefly, the olfactometer consisted of five zones: (1) Central zone: Connected to a vacuum pump with an airflow of 600 mL/min to direct volatile compounds toward the center. This is where the insects were placed for the assay. (2) Stimulus arms: Two arms located opposite each other where glass columns were inserted. Filter papers (Whatman No. 1, 0.3 cm wide × 4 mm long) impregnated with 10 µL of volatiles were placed in these arms. First, the attraction to the plant extracts obtained previously (see Section 2.2) was evaluated, focusing on the wild ancestor ecotypes and their offspring (domesticated ecotypes). In the initial olfactometric assays, we evaluated whole-plant extracts that contained complex mixtures of volatile organic compounds from both wild and cultivated ecotypes. These extracts provided a volatile profile representative of natural conditions, allowing us to assess insect behavioral responses to the overall VOC emission spectrum of each ecotype. Subsequently, individual pure compounds were tested at four different concentrations (0.1, 1, 10, and 100 ppm) to evaluate dose-dependent behavioral responses.

The concentrations of volatile compounds were selected based on concentrations previously reported and commonly used to assess insect attraction to volatile compounds [39,40,41,42,43]. Moreover, the volatiles tested included compounds identified by GC-MS analysis, categorized as follows: (A) Alcohols and ketones: 2-hexanol, 3-hexanol, 2-hexanone, 3-hexanone, and 2,4-dimethyl acetophenone, and (B) terpenes: 1,8-cineole, S-limonene, R-limonene, sabinene, α-pinene (+ and −), β-myrcene, caryophyllene, and α-caryophyllene. (3) Control arms: The remaining two arms contained filter papers impregnated with hexane serving as the control. The insects’ behavior was monitored based on the time spent in each of the five areas of the olfactometer (n = 30 insects per trial). A stopwatch was used to record the residence time of each insect. Additionally, an olfactometric preference index (OPI) was calculated to assess the insects’ attraction to the stimuli (extracts and compounds). The OPI was calculated using the following formula:

$$\mathrm{OPI}=\frac{2S}{S+C}$$

where S represents the time spent in the stimulus area and C the time in the control area. OPI values range from 0 to 2, with a value of 1 indicating no preference, values less than 1 indicating a preference for the control, and values greater than 1 indicating a preference for the stimulus [40,41].

### 2.5. Y-Tube Olfactometric Assays

For *C. defreitasi* adult lacewings, Y-tube olfactometric assays were conducted to evaluate their response to the same volatile compounds used in the 4-arm assays. The Y-tube was positioned horizontally, and clean air flowed at 600 mL/min through the base of the tube. In one arm, filter paper with 10 µL of volatile compounds (alcohols, ketones, and terpenes) was placed at the same concentrations used in the 4-arm assays. On the opposite arm, a control filter paper containing 10 µL of hexane was placed. An individual lacewing adult was introduced into the base of the Y-tube, and its response was categorized into two options: (1) C: preference for the control and (2) S: preference for the stimulus. Each compound and concentration were tested 60 times (n = 60), and each repetition was evaluated for 40 min [42,43]. This olfactometer was selected specifically due to the size of the adult lacewings and, more importantly, their ability to fly, in contrast to the larvae, which are walking organisms. Therefore, the Y-tube olfactometer proved optimal for evaluating the behavioral response of adult lacewings, as it allowed for proper assessment of their flight behavior and orientation toward the volatile compounds.

### 2.6. Statistical Analysis

Statistical analyses were conducted using Statistix 10 software (Analytical Software, Tallahassee, FL, USA). To determine differences in the total concentration of volatile organic compounds between all wild and domesticated ecotypes, a *t*-test was performed. Then, to determine the variability of each compound individually among the different ecotypes, an analysis of variance (ANOVA) followed by Tukey’s test was performed. To observe the patterns of VOCs based on their abundance and classification, as well as to analyze their impact on the degree of domestication in murtilla, a principal component analysis (PCA) was performed for each case. Subsequently, hierarchical cluster analysis was conducted to determine the degree of similarity or grouping among the different individuals or groups evaluated based on their chemical profiles. The similarity index used was the Pearson correlation coefficient, and clustering was performed using the Ward’s method. For the 4-arm olfactometric assays, ANOVA followed by Tukey’s test was performed to analyze the effects of extracts and pure compounds on insect behavior. Prior to ANOVA, data normality was verified using the Shapiro–Wilk test, and the homogeneity of variances was assessed using Levene’s test. For the Y-tube olfactometric assays, we analyzed the insect choice data using chi-square tests (goodness of fit), assuming a Poisson distribution of independent events, to determine whether lacewing adult preferences for specific odors deviated significantly from a 1:1 distribution. Statistical significance was set at *p* ≤ 0.05. Results are presented as means with their corresponding standard errors in figures and as means with standard deviations in tables.

## 3. Results

### 3.1. Comparison of VOC Profiles Between Domesticated and Wild Murtilla Plants

**Figure 1:**
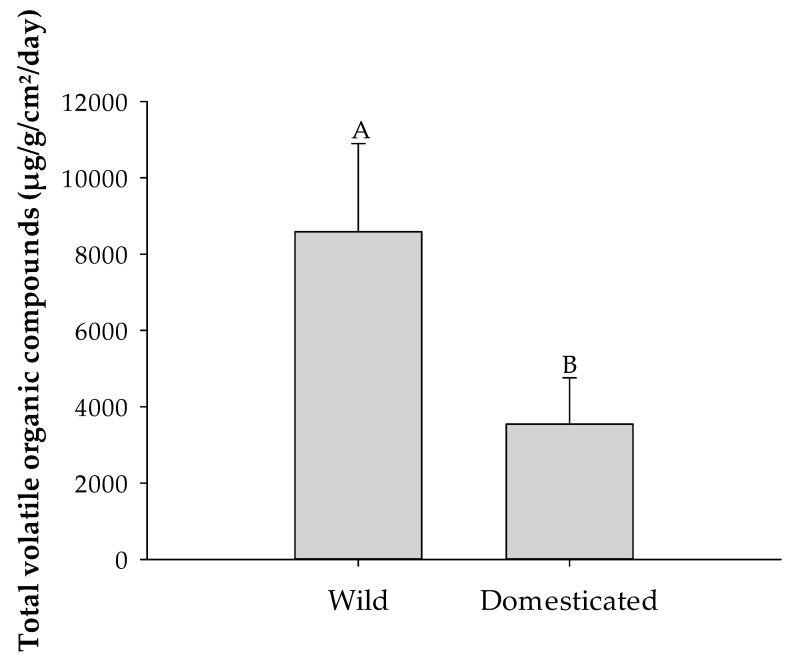
Total emission of volatile organic compounds in wild and domesticated *Ugni molinae* plants (µg/g/cm^2^/day). Different letters indicate statistically significant differences between groups according to the *t*-test (*p* < 0.05). Bars represent means ± SE.

Figure 1 shows a significant difference in the total emission of VOCs between wild and domesticated *U. molinae* plants (t_2.1_ = 5.83, *p* = 0.0241), with wild plants emitting significantly higher levels of volatiles than domesticated ones. In contrast, Table 2 presents the results of individual analyses for each volatile compound, comparing their concentrations across the different ecotypes. These compound-level comparisons indicate that even early stages of domestication, represented by selected F1 progeny from controlled crosses, can lead to significant variation in the emission levels of specific VOCs among ecotypes (Table 3). For instance, key compounds such as 2-hexanone and α-caryophyllene were substantially reduced or even undetectable in domesticated progeny compared to wild ecotypes. Similar trends were observed for other volatiles, including 3-hexanone, 3-hexanol, and 2-hexanol, with the highest concentrations consistently found in wild ecotypes and the lowest in domesticated lines. Wild plants also showed markedly higher levels of monoterpenes such as pinene, sabinene, β-myrcene, and 1,8-cineole, as well as sesquiterpenes such as caryophyllene. These reductions in volatile emissions suggest a clear trend driven by domestication, with potential ecological consequences for plant–insect interactions mediated by these chemical defenses.

### 3.2. Distribution and Variability of Volatile Compounds in Ugni molinae Plants

**Figure 2:**
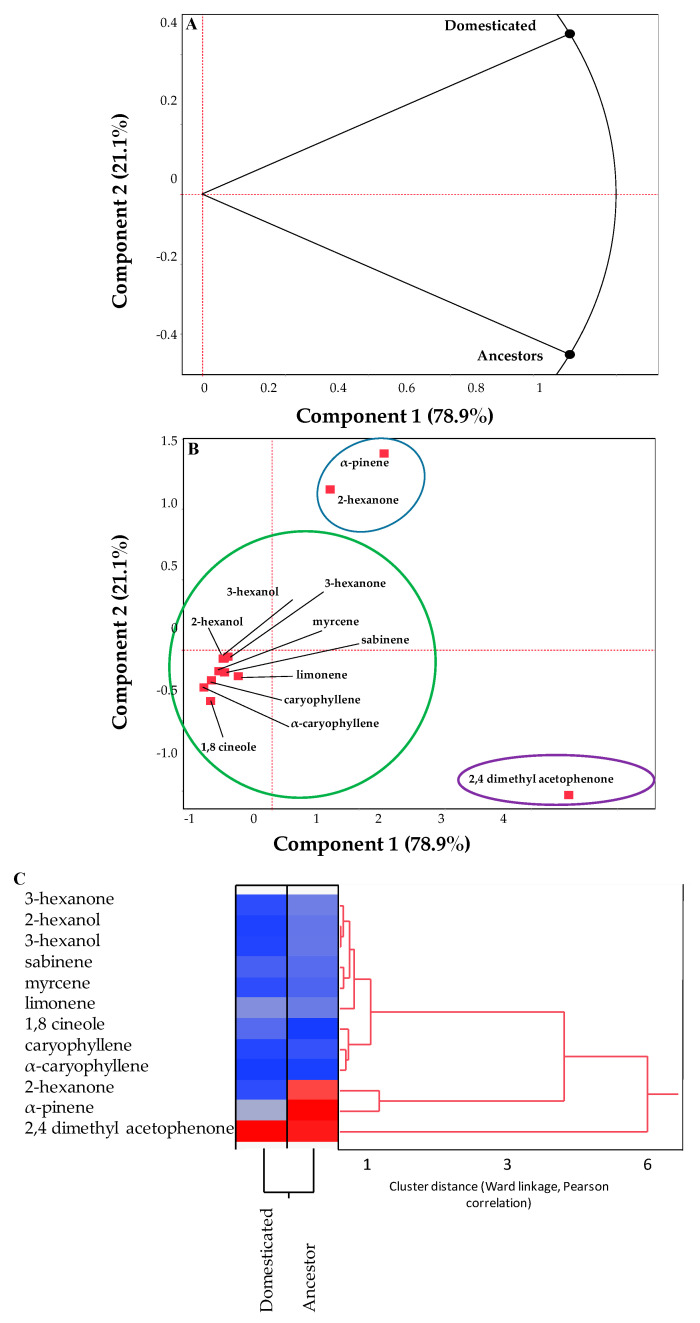
Principal component analysis of volatile organic compounds in cultivated ecotypes and wild ancestors of murtilla. (**A**) Separation between domesticated ecotypes and ancestors based on the first two principal components. (**B**) Distribution of VOCs according to their contribution to the total variability. (**C**) Hierarchical cluster analysis (HCA) of volatile organic compounds in murtilla. The heatmap shows the relative abundance of each compound (blue: low abundance, red: high abundance). The dendrogram groups both samples and compounds according to chemical similarity, using Ward’s linkage and Pearson correlation as the similarity index. A relative distance scale is shown at the bottom of the dendrogram.

In the PCA results (Figure 2), Component 1 explains 78.9% of the variation, while Component 2 accounts for 21.1%, capturing most of the variance in the dataset. Figure 2A highlights a clear separation between domesticated ecotypes and their wild ancestors, emphasizing differences in volatile profiles. This suggests that domestication processes significantly influenced the composition and abundance of VOCs. Figure 2B details the relationship between specific VOCs and the two groups (domesticated and ancestors). Compounds such as 2-hexanone and α-pinene are closely associated with domesticated ecotypes, suggesting these volatiles may be characteristic markers of domestication. In contrast, sesquiterpenes such as caryophyllene and α-caryophyllene, along with 1,8-cineole, appear to be more abundant or characteristic of the ancestral group. Additionally, 2,4-dimethyl acetophenone, a unique ketone, stands out as an outlier in the PCA, indicating that its presence is less correlated with the major clusters.

The heatmap (Figure 2C) illustrates the relative abundance of the VOCs (red for higher abundance and blue for lower abundance), while the dendrogram shows the hierarchical clustering of the compounds and samples. For the heatmap, the color gradient from blue to red represents the variability in the abundance of VOCs among the samples. Specific compounds with higher intensities are more associated with particular groups, indicating their prominence in certain ecotypes (domesticated or wild ancestors). Conversely, compounds with lower abundance are less characteristic of those groups. For the dendrogram, the hierarchical clustering identifies two main groups. The clustering reveals strong associations between VOCs with similar chemical or biosynthetic pathways. The structure indicates that certain compounds share similar abundance patterns, further distinguishing domesticated ecotypes from wild relatives. This analysis supports the differentiation observed in the PCA, confirming that the volatile profiles of domesticated and ancestral murtilla samples are distinct, with specific clusters of VOCs defining these groups. Therefore, the heatmap reveals distinct differences in volatile compound composition between ancestral and domesticated ecotypes.

Domesticated ecotypes are characterized by a higher presence of 2,4-dimethyl acetophenone and α-pinene (red zones), while ancestral ecotypes show higher abundances of 1,8-cineole, limonene, sabinene, myrcene, caryophyllene, and various hexanones and hexanols (blue zones). This pattern suggests that domestication resulted in a reduction in the diversity of certain terpenes and alcohols while increasing the presence of specific ketones.

### 3.3. Olfactometric Preference Index for Ugni molinae Extracts in Lacewing Larvae and Aphid Adults

**Figure 3:**
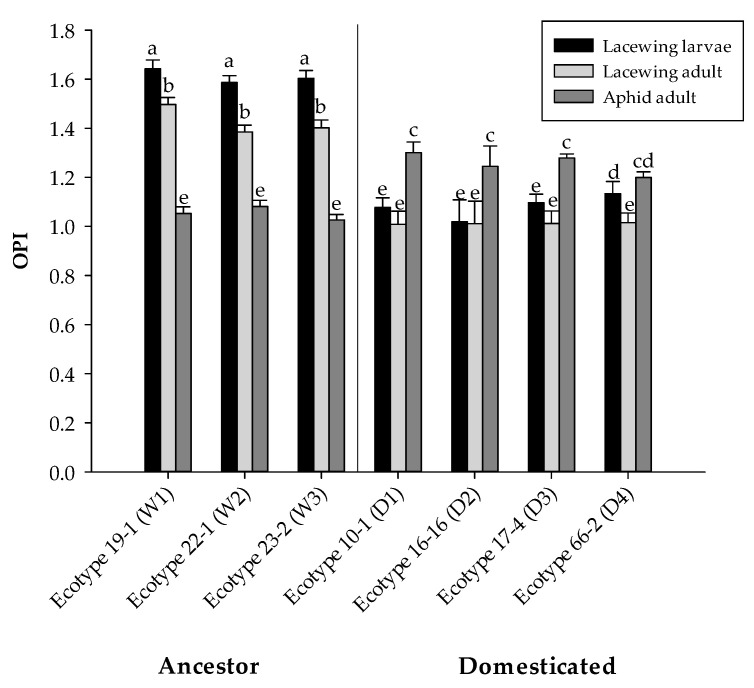
Olfactometric preference index (OPI) of different ecotypes in ancestor and domesticated groups when exposed to lacewing larvae, lacewing adults, and aphid adults. Different letters indicate significant differences (*p* < 0.05) among treatments. Bars represent means ± SE.

As shown in Figure 3, ancestral ecotypes such as Ecotype W1 exhibited the highest attraction for lacewing larvae (1.64 ± 0.03 OPI) and adults (1.49 ± 0.02 OPI), while aphid adults displayed the lowest OPI (1.05 ± 0.02 OPI). Similarly, Ecotype W2 and Ecotype W3 exhibited high attraction for lacewing larvae (1.58 ± 0.02 and 1.60 ± 0.03 OPI, respectively) and adults (1.38 ± 0.02 and 1.40 ± 0.03 OPI), with aphids maintaining lower values (1.08 ± 0.02 and 1.02 ± 0.02 OPI). In the domesticated ecotypes, the trend shifts, with Ecotype D1, Ecotype D2, Ecotype D3, and Ecotype D4 showing significantly lower attraction for lacewing larvae (ranging from 1.01 ± 0.09 to 1.13 ± 0.04 OPI) and adults (1.00 ± 0.05 to 1.01 ± 0.03 OPI), while aphids had comparatively higher OPI values, particularly in Ecotype D1 (1.30 ± 0.04 OPI) and Ecotype D3 (1.27 ± 0.01 OPI). These differences in insect attraction across ecotypes and ecotype types (wild vs. domesticated) were statistically significant, as confirmed by ANOVA, followed by Tukey’s test (F_12,399_ = 20.83, *p* < 0.0001).

### 3.4. Olfactometric Responses of Aphids and Lacewings in a 4-Arm Olfactometer to Pure Compounds

**Figure 4:**
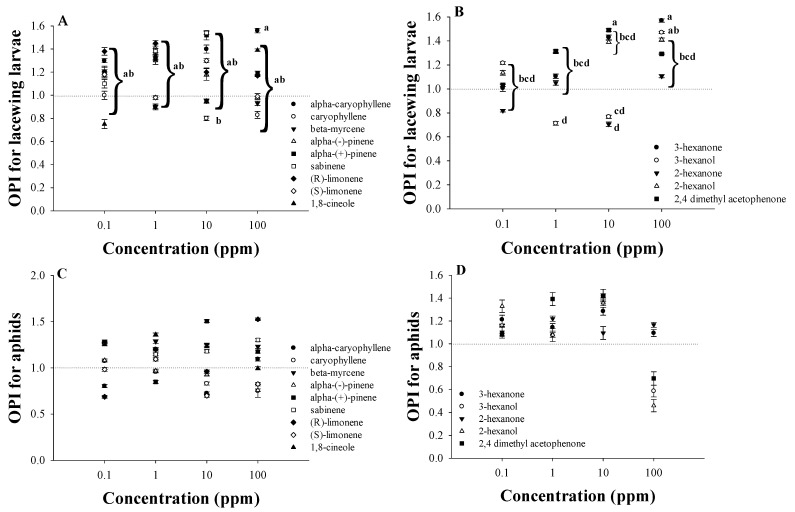
Olfactometric preference index (OPI) for lacewing larvae and terpenes (**A**) and alcohols, ketones and esters (**B**), OPI for aphid and terpenes, (**C**) and alcohols, ketones, and esters (**D**). Different letters mean significant differences according to ANOVA analysis followed by a Tukey test (*p* ≤ 0.05). Points represent means ± SE.

From 1440 assays (12 compounds, 4 concentrations, and 30 repetitions), the olfactometric responses showed that compounds significantly influenced the olfactory preference index (OPI) for both aphids and lacewing larvae (F_11,1428_ = 3.16, *p* ≤ 0.001; F_11,1428_ = 3.15, and *p* ≤ 0.001, respectively) (Figure 4). However, for the concentration of compounds, the results show no significant influence on the response of aphids or lacewing larvae (F_3,1424_ = 0.31, *p* = 0.816; F_3,1424_ = 1.33, *p* = 0.260, respectively). Regarding the parallelism test (compound × concentration), our results show that for aphids, no significant influence on the insect’s response was observed (F_33,1391_ = 1.43, *p* = 0.082). However, for lacewing larvae, the interaction had a significant influence (F_33,1391_ = 1.76, *p* = 0.013). For instance, lacewing larvae preferred α-caryophyllene with an OPI of 1.6 at 100 ppm (Figure 4A). For a focused analysis on alcohols, ketones, and esters (five selected compounds), our results indicate that for lacewing larvae, compound, concentration, and their interaction significantly influenced the larvae’s response (F_4,579_ = 6.96, *p* ≤ 0.001; F_3,579_ = 6.32, *p* ≤ 0.001; F_12,579_ = 2.62, and *p* ≤ 0.001, respectively). Figure 4B shows a significant increase in OPI for 2,4-dimethyl acetophenone at 10 ppm and for 3-hexanone and 3-hexanol at 100 ppm, with OPI values of 1.5, 1.6, and 1.5, respectively. Finally, for aphids, OPI was significantly influenced by concentration, while compounds and the interaction (compound × concentration) were not significant (F_4,579_ = 0.64, *p* = 0.628; F_3,579_ = 10.31, *p* ≤ 0.001; F_12,579_ = 1.39, and *p* = 0.166, respectively) (Figure 4C,D).

### 3.5. Lacewing Adult Responses to Volatiles in a Y-Tube Olfactometer to Pure Compounds

From 2880 olfactometric assays in a Y-tube (12 compounds, 4 concentrations, and 60 repetitions), the results show that the preference of lacewing adults was significantly influenced by the compounds, concentration, and their interaction (X^2^_(11)_ = 412.65, *p* ≤ 0.001; X^2^_(3)_ = 62.14, *p* ≤ 0.001; X^2^_(47)_ = 91.27, and *p* = 0.001, respectively) (Table 4). The results indicate that the attraction of lacewing adults varied depending on the compound and concentration tested. Among the terpenes, α-(−)-pinene induced a significant preference for the stimulus at all concentrations, with attraction values of 86.7% at 0.1 ppm and reaching 100% at 100 ppm. A similar pattern was observed for α-(+)-pinene, where a preference for the stimulus was significant at all concentrations, peaking at 96.7% at 100 ppm. In contrast, (R)-limonene did not show significant differences at any concentration, with responses being more balanced between the control and the stimulus. For sabinene, a significant preference for the stimulus was recorded only at 1 ppm (66.7%), while at the remaining concentrations, responses were similar between the control and stimulus. (S)-limonene exhibited a strong attraction to the stimulus at 0.1 ppm (63.4%), 10 ppm (73.4%), and 100 ppm (80.0%). β-Myrcene showed significant variation in response, with higher attraction to the control at 0.1 ppm (70%) and 1 ppm (60%), but a preference for the stimulus at 10 ppm (73.4%) and 100 ppm. Caryophyllene elicited a significant preference for the stimulus at 1 ppm (70%), increasing to 90% at the highest concentration tested. Lastly, α-caryophyllene generated strong attraction at all concentrations, with preference values of 76.7% at 100 ppm. In the group of alcohols, ketones, and esters, both compounds and concentration significantly influenced lacewing responses (X^2^_(4)_ = 31.98, *p* = 0.002; X^2^_(3)_ = 45.61, and *p* ≤ 0.001, respectively), but their interaction did not (X^2^_(12)_ = 28.34, *p* = 0.127). Larval responses also varied depending on the compound and concentration. 2-hexanol exhibited a significant and increasing trend of attraction to the stimulus, reaching 76.7% at 10 ppm and 70.0% at 100 ppm. Similarly, 3-hexanone elicited a significant response toward the stimulus starting at 1 ppm, with values increasing to 86.7% at the highest concentration. 3-hexanol induced a preference for the stimulus at all concentrations, with 73.4% at 10 ppm and 80% at 100 ppm. 2,4-Dimethyl acetophenone also induced consistent attraction, with the highest preference observed at 1 ppm (90.0%), 10 ppm (90.0%), and 100 ppm (93.4%). Finally, 2-hexanone followed a similar trend to other compounds in its group, with a significant preference for the stimulus starting at 1 ppm (73.4%) and reaching 90% at 100 ppm. Overall, the results suggest that certain terpenes, such as α-(−)-pinene and α-caryophyllene, strongly attracted lacewing adults, whereas other compounds, such as (R)-limonene, did not elicit significant effects. Among alcohols, ketones, and esters, several compounds showed a clear trend of preference for the stimulus, with attraction increasing as concentration increased.

## 4. Discussion

The findings of this study provide significant insights into the impact of domestication on the chemical ecology of murtilla, specifically regarding the emission of volatile organic compounds and their influence on plant–insect interactions. Our results indicate that domestication led to a marked reduction in the emission of key defensive VOCs, thereby altering the plant’s natural defense mechanisms against herbivores and predators. This aligns with the broader plant domestication defense theory, which suggests that the selection for agronomic traits often comes at the expense of natural resistance to pests and diseases [1,2,3,4,5]. A key observation in our study is that ancestors of murtilla emit significantly higher levels of volatile organic compounds compared to domesticated ones. These emissions include compounds known to attract predatory insects, such as lacewing larvae, which are essential biological control agents in natural ecosystems. Our gas chromatography analysis identified several compounds, including 2-hexanone, 1,8-cineole, and α-caryophyllene, that were emitted in greater concentrations by murtilla ancestors. These findings are consistent with previous studies on other domesticated plants, such as wild tomatoes and maize, where domestication similarly led to a reduction in the emission of herbivore-induced plant volatiles, subsequently decreasing attraction to natural enemies of herbivores [8,9,24,25]. Our results reinforce this pattern, showing that domesticated *U. molinae* plants emit significantly lower amounts of key defensive volatiles, which may influence plant–insect interactions and potentially have profound implications for pest management and ecological stability [44,45]. A key aspect of the plant domestication defense theory is the shift in resource allocation, where plants selected for higher yield or better fruit quality often exhibit reduced investment in chemical defenses. In the case of *U. molinae*, the observed decline in VOC emissions in domesticated plants suggests that selection pressures favored traits beneficial for cultivation at the expense of defense-related metabolites. This is in line with the study by Chacón-Fuentes et al. [5], which reveals significant differences in VOC production between wild ecotypes and cultivated murtilla. The wild relatives showed a higher emission rate of 624.6 µg/cm^2^/day, while the domesticated plants emitted considerably lower levels, with an average emission of approximately 439.3 µg/cm^2^/day under herbivory conditions. This highlights the ecological consequences of artificial selection, where breeding for agronomic traits may inadvertently disrupt natural defense strategies evolved over millennia [46]. The decreased attraction of lacewing larvae to domesticated plants suggests that the loss of VOCs not only affects direct plant–herbivore interactions, but also disrupts indirect defense mechanisms. Lacewings and other natural predators rely on volatile cues to locate their prey, and reductions in these signals may impair the efficiency of biological control in agricultural settings [47,48]. Previous studies have shown that many predatory insects use plant volatiles as foraging cues, particularly those released in response to herbivore damage [49,50]. The diminished recruitment of lacewings in domesticated *U. molinae* may therefore reflect a broader trend where domestication reduces the effectiveness of natural enemies, increasing reliance on synthetic pesticides.

Our olfactometric preference assays further support these chemical analyses by demonstrating that lacewing larvae exhibited a stronger preference for VOCs emitted by wild ecotypes, whereas aphids showed greater attraction to domesticated ecotypes. The olfactometric preference index confirmed that wild ecotypes had a higher likelihood of attracting lacewing larvae, which are natural aphid predators, while domesticated plants were more susceptible to aphid infestation due to their reduced volatile emissions. Bioassays with pure compounds further validated these results, showing that lacewing larvae were strongly attracted to α-caryophyllene and 2,4-dimethyl acetophenone, both of which were present in significantly higher concentrations in wild ecotypes. Conversely, aphids were more attracted to compounds such as 2-hexanone and 3-hexanol, which were emitted at higher levels by domesticated ecotypes. These findings suggest that the specific VOC profile of murtilla plays a crucial role in mediating plant–insect interactions and that the reduction in these compounds due to domestication directly influenced the plant’s vulnerability to herbivores. The increased preference of aphids for domesticated plants further supports this, suggesting that the reduction in deterrent compounds made them more vulnerable to infestation. This shift in herbivore preference could have significant agronomic implications, particularly in commercial production systems where aphid infestations contribute to crop losses and increased disease transmission [51]. From an ecological perspective, the observed changes in plant–insect interactions highlight the broader consequences of domestication on trophic networks [52]. The reduction in defensive VOCs not only affects herbivores and predators, but also has potential cascading effects on other organisms that depend on plant volatiles for communication, such as pollinators and microbial communities [53]. In natural ecosystems, VOCs serve multiple functions beyond herbivore deterrence, including attracting pollinators and mediating plant–plant signaling [54]. The loss of these compounds in domesticated *U. molinae* could therefore have unintended effects on ecosystem dynamics, potentially reducing pollinator visitation rates and altering microbial associations that contribute to plant health and soil fertility. One of the key challenges in modern agriculture is balancing productivity with ecological resilience [55]. The findings of this study suggest that breeding programs should consider reintegrating lost defense traits to mitigate the unintended consequences of domestication. Several strategies could help restore VOC-mediated defenses in *U. molinae* without compromising yield.

In summary, this study provides compelling evidence that domestication significantly altered the chemical defenses of *U. molinae*, reducing its ability to deter aphids and attract natural predators. Our findings strongly support the plant domestication defense theory, demonstrating that domesticated *U. molinae* reduced VOC emissions, increasing susceptibility to aphids while reducing the attraction of natural predators. This research underscores the need to integrate ecological principles into breeding programs to balance productivity and pest resistance. Overall, our study highlights the complex interplay between domestication, chemical defenses, and plant–insect interactions. By demonstrating that domesticated murtilla lost key VOC-mediated defenses, we emphasize the potential risks associated with breeding practices that prioritize fruit yield and quality over natural resistance mechanisms. The increased susceptibility of domesticated *U. molinae* to herbivores and the reduced recruitment of natural enemies underscore the ecological costs of domestication.

Although our findings provide strong evidence that early selection in murtilla is associated with reductions in defensive volatile organic compounds, it is important to note that the genotypes analyzed represent the selected F1 progeny derived from a limited pool of wild parental ecotypes. As such, they reflect a specific and early stage in the broader domestication process. Future studies that include a wider diversity of domesticated germplasm, including advanced generations and independent selection lines, will be essential to confirm the generality of these results and further elucidate the evolutionary trajectory of plant defenses during domestication.

Future studies should explore restoring VOC-mediated defenses through selective breeding, microbial inoculants, or intercropping strategies, aiming to develop sustainable pest management solutions for *U. molinae* and other cultivated species. Our findings contribute to a growing body of evidence that highlights the need for more holistic breeding strategies and balancing agronomic improvement with ecological resilience. By incorporating ecological principles into crop improvement strategies, it may be possible to develop sustainable cultivation systems that optimize yield while enhancing pest resilience, ensuring the long-term viability of *U. molinae* as a cultivated species. Future research should continue exploring ways to integrate natural plant defenses into modern crop breeding, ensuring that domesticated plants retain the ability to interact beneficially with their ecological surroundings while maintaining productivity and market viability.

## 5. Conclusions

This study provides valuable insights into how domestication affects the volatile organic compound profile of murtilla, influencing its interactions with herbivores and predators. Our results demonstrate that domesticated murtilla plants emit significantly lower levels of key VOCs compared to their wild counterparts, as measured through chromatography. Ancestor ecotypes of murtilla exhibited a more diverse array of VOCs, including 2-hexanone, 1,8-cineole, and α-caryophyllene, which play crucial roles in attracting natural predators, such as lacewing larvae, and deterring herbivores, such as aphids. In contrast, domesticated plants showed a reduction in the diversity and concentration of these compounds. Bioassays with pure compounds revealed that these reduced VOC levels in domesticated plants led to a diminished attraction of lacewing larvae and increased preference of aphids. These findings suggest that domestication compromised the plant’s ability to recruit beneficial predators and protect itself from pests. The reduction in defensive VOCs highlights the ecological consequences of domestication and underscores the need for future breeding strategies to consider the preservation of natural defense mechanisms alongside productivity goals.

## Figures and Tables

**Table 1:** Origin of the domesticated and the wild ancestor ecotypes involved in their breeding.

Domesticated Plants	Wild Ancestors
Ecotype 10-1 (D1 *)	Ecotypes 22-1 (W2) × 19-1
Ecotype 16-16 (D2)	Ecotypes 19-1 (W1) × 22-1
Ecotype 17-4 (D3)	Ecotypes 23-2 (W3) × 22-1
Ecotype 66-2 (D4)	Ecotypes 23-2 × 19-1

* indicates the abbreviation used hereafter in the text to refer to this ecotype.

**Table 2:** Quantification of volatile compounds in different *Ugni molinae* ecotypes. The table presents the mean concentrations (±standard deviation) of alcohols, ketones, monoterpenes, and sesquiterpenes across seven ecotypes. Different letters indicate statistically significant differences among ecotypes for each compound (*p* < 0.05). ND = Not detected.

Compounds (µg/g/cm^2^/day)		W1	W2	W3	D1	D2	D3	D4
Alcohols and ketones	2-Hexanone	12.71 ± 0.68 ^b^	11.82 ± 0.95 ^b^	15.31 ± 0.41 ^a^	7.10 ± 0.31 ^c^	6.46 ± 0.62 ^c^	5.53 ± 0.10 ^d^	5.91 ± 0.72 ^d^
	3-Hexanone	6.12 ± 0.53 ^ab^	7.81 ± 1.25 ^a^	5.79 ± 0.32 ^b^	1.81 ± 0.21 ^d^	2.71 ± 0.11 ^c^	0.52 ± 0.11 ^e^	2.72 ± 1.81 ^c^
	3-Hexanol	5.31 ± 0.71 ^b^	7.47 ± 0.42 ^a^	4.47 ± 0.21 ^c^	1.21 ± 0.47 ^e^	1.72 ± 0.31 ^d^	0.63 ± 0.01 ^f^	1.60 ± 0.71 ^d^
	2-Hexanol	7.02 ± 0.61 ^b^	6.49 ± 0.73 ^c^	10.26 ± 1.01 ^a^	2.23 ± 0.34 ^b^	2.54 ± 0.02 ^e^	0.86 ± 0.02 ^f^	2.96 ± 0.63 ^d^
	2,4-Dimethyl acetophenone	86.11 ± 2.56 ^a^	83.63 ± 2.41 ^a^	68.16 ± 5.12 ^b^	43.57 ± 8.47 ^d^	53.51 ± 8.47 ^c^	48.39 ± 4.45 ^cd^	32.15 ± 4.11 ^f^
Monoterpenes	Pinene	12.65± 1.22 ^b^	29.91 ± 6.62 ^a^	13.88 ± 3.36 ^b^	9.49 ± 0.42 ^d^	9.12 ± 1.13 ^d^	10.10 ± 0.97 ^c^	8.30 ± 0.80 ^e^
	Sabinene	12.49 ± 0.2 ^b^	15.10 ± 1.80 ^a^	10.65 ± 0.52 ^c^	2.41 ± 0.91 ^d^	0.11 ± 0.02 ^e^	0.22 ± 0.08 ^e^	0.12 ± 0.01 ^e^
	β-Myrcene	6.18 ± 0.02 ^c^	18.70 ± 2.02 ^a^	15.21 ± 0.10 ^b^	ND	0.10 ± 0.01 ^c^	0.21 ± 0.06 ^c^	0.11 ± 0.01 ^c^
	1,8 Cineole	7.62 ± 0.02 ^b^	19.31 ± 0.21 ^a^	7.51 ± 0.02 ^b^	0.10 ± 0.02 ^c^	0.21 ± 0.02 ^c^	0.21 ± 0.03 ^c^	0.11 ± 0.01 ^c^
	Limonene	7.30 ± 0.3 ^b^	11.72 ± 1.90 ^a^	0.59 ± 0.11 ^c^	0.51 ± 0.02 ^c^	0.52 ± 0.01 ^c^	0.20 ± 0.01 ^d^	0.11 ± 0.01 ^d^
Sesquiterpenes	Caryophyllene	0.41 ± 0.0 ^b^	1.18 ± 1.21 ^a^	0.27 ± 0.02 ^c^	ND	ND	0.31 ± 0.08 ^c^	0.10 ± 0.01 ^d^
	α-Caryophyllene	0.29 ± 0.01 ^b^	1.54 ± 0.31 ^a^	0.13 ± 0.11 ^c^	ND	ND	ND	0.11 ± 0.01 ^c^

**Table 3:** Summary of ANOVA results for volatile compound concentrations across seven *Ugni molinae* ecotypes.

Compound Family	Compound	Degree of Freedom	Error	Total	F-Value	*p*-Value
Alcohols and ketones	2-Hexanone	6	56	62	23.86	0.0000
	3-Hexanone	6	56	62	4.06	0.0019
	3-Hexanol	6	56	62	25.44	0.0000
	2-Hexanol	6	56	62	5.35	0.0002
	2,4-Dimethyl acetophenone	6	56	62	4.10	0.0018
Monoterpenes	Pinene	6	56	62	4.43	0.0010
	Sabinene	6	56	62	9.31	0.0000
	β-Myrcene	6	56	62	5.08	0.0003
	1,8 Cineole	6	56	62	2.98	0.0134
	Limonene	6	56	62	3.01	0.0126
Sesquiterpenes	Caryophyllene	6	56	62	9.33	0.0000
	α-Caryophyllene	6	56	62	2.28	0.0489

**Table 4:** Percentages of preference for lacewing adults in relation to different compounds and concentrations. C = control treatments and S = stimuli treatment. * mean significant differences according to chi-square test.

Concentration (ppm)		0.1		1		10		100	
Response (%)		C	S	C	S	C	S	C	S
Compounds									
	Terpenes								
1,8 cineole		53.3	46.7	40.0	60.0 *	60.0 *	40.0	16.6	83.4 *
(*S*) limonene		36.6	63.4 *	60.0 *	40.0	26.6	73.4 *	20.0	80.0 *
(*R*) limonene		70.0 *	30.0	50.0	50.0	36.6	63.4 *	43.3	56.7
sabinene		50.0	50.0	33.3	66.7 *	43.3	56.7	43.3	56.7
α-(−)-pinene		13.3	86.7 *	20.0	80.0 *	10.0	90.0 *	0.0	100.0 *
α-(+)-pinene		23.3	76.7 *	23.3	76.7 *	20.0	80.0 *	3.33	96.7 *
β-myrcene		70.0 *	30.0	60.0 *	40.0	46.7	53.3	26.6	73.4 *
caryophyllene		43.3	56.7	30.0	70.0 *	36.6	63.4 *	10.0	90.0 *
α-caryophyllene		63.3 *	36.7	53.3	46.7	33.3	66.7 *	23.3	76.7 *
	Alcohols, ketones, and esters								
2-hexanol		53.3	46.7	33.3	66.7 *	23.3	76.7 *	30.0	70.0 *
3-hexanone		50.0	50.0	33.3	66.7 *	20.0	80.0 *	13.3	86.7 *
3-hexanol		33.3	66.7 *	33.3	66.7 *	16.6	73.4 *	20.0	80.0 *
2,4 dimethyl acetophenone		26.6	73.4 *	10.0	90.0 *	10.0	90.0 *	6.6	93.4 *
2-hexanone		43.3	56.7	26.6	73.4 *	16.6	83.4 *	10.0	90.0 *

## Data Availability

The original contributions presented in this study are included in the article. Further inquiries can be directed to the corresponding authors.
